# Lysine 2‐Hydroxyisobutyrylation‐ and Succinylation‐Based Pathways Act Inside Chloroplasts to Modulate Plant Photosynthesis and Immunity

**DOI:** 10.1002/advs.202301803

**Published:** 2023-07-26

**Authors:** Bin Chen, Zhicheng Wang, Mengjia Jiao, Jin Zhang, Jie Liu, Dongmei Zhang, Yanbin Li, Guoning Wang, Huifeng Ke, Qiuxia Cui, Jun Yang, Zhengwen Sun, Qishen Gu, Xingyi Wang, Jinhua Wu, Liqiang Wu, Guiyin Zhang, Xingfen Wang, Zhiying Ma, Yan Zhang

**Affiliations:** ^1^ State Key Laboratory of North China Crop Improvement and Regulation North China Key Laboratory for Germplasm Resources of Education Ministry Hebei Agricultural University Baoding 071001 China

**Keywords:** chloroplast, cotton, crop yield, disease resistance, epigenetic regulation

## Abstract

Crops must efficiently allocate their limited energy resources to survival, growth and reproduction, including balancing growth and defense. Thus, investigating the underlying molecular mechanism of crop under stress is crucial for breeding. Chloroplasts immunity is an important facet involving in plant resistance and growth, however, whether and how crop immunity modulated by chloroplast is influenced by epigenetic regulation remains unclear. Here, the cotton lysine 2‐hydroxyisobutyrylation (Khib) and succinylation (Ksuc) modifications are firstly identified and characterized, and discover that the chloroplast proteins are hit most. Both modifications are strongly associated with plant resistance to *Verticillium dahliae*, reflected by Khib specifically modulating PR and salicylic acid (SA) signal pathway and the identified GhHDA15 and GhSRT1 negatively regulating Verticillium wilt (VW) resistance via removing Khib and Ksuc. Further investigation uncovers that photosystem repair protein GhPSB27 situates in the core hub of both Khib‐ and Ksuc‐modified proteins network. The acylated GhPSB27 regulated by GhHDA15 and GhSRT1 can raise the D1 protein content, further enhancing plant biomass‐ and seed‐yield and disease resistance via increasing photosynthesis and by‐products of chloroplast‐derived reactive oxygen species (cROS). Therefore, this study reveals a mechanism balancing high disease resistance and high yield through epigenetic regulation of chloroplast protein, providing a novel strategy to crop improvements.

## Graphical abstract

The cotton lysine 2‐hydroxyisobutyrylation and succinylation modifications are firstly identified. Both modifications are strongly associated with plant resistance to *Verticillium dahliae*. Further investigation uncovers that GhPSB27 situates in the core hub of modified proteins network and regulated by GhHDA15 and GhSRT1 can raise the D1 protein content, further enhancing plant yield and disease resistance via increasing photosynthesis and ROS.

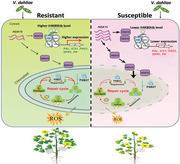

## 1 Introduction

Unlike animals, crops lack an adaptive immune system for recognizing and eliminating invading pathogens actively. Therefore, plants have evolved a set of sophisticated regulatory mechanisms for defensing against external stresses.^[^ ^1^ ^]^ For instance, when attacked by pathogens, plant growth is usually slowed by an active response that transfers more energy resources to activate the defense system, resulting in yield penalties, whereas, they can resume growth once the threat is averted.^[^ ^2^, ^3^ ^]^ Crops without an active immune response may grow significantly faster but will more easily succumb to the diseases. Breeding crops with resistance is an efficient way to control diseases. However, increased resistance often comes with an unintended reduction in growth and yield. To optimize fitness, plants must efficiently allocate their energy resources between growth and defense.

High yield and immunity to pathogens are crucial objectives but challenging in crop breeding. In details, plants defeating pathogens often depend on the interplay between different hormone systems, such as salicylic acid (SA), jasmonic acid (JA), ethylene, and auxin.^[^ ^4^ ^]^ At the same time, the phytohormones also participate in regulating multiple aspects of plant development.^[^ ^5^ ^]^ Thus, the signaling cross‐talks from diversified hormone networks often display antagonistic effect in growth and defense. For instance, SA‐mediated disease‐resistance mechanism partly inhibited the auxin signaling pathway, reducing growth via stabilizing the Aux/IAA repressor proteins.^[^ ^6^ ^]^ In maize, the *ZmAuxRP1* functioned as a promoter in the biosynthesis of indole‐3‐acetic acid, whereas it simultaneously suppressed the formation of defense compound benzoxazinoids.^[^ ^7^ ^]^ In rice, overexpressing *OsNPR1* greatly enhanced resistance to bacterial blight by sacrificing the growth and development.^[^ ^8^ ^]^ Another signaling balancing growth and defense involved the cross‐talk between JA and gibberellin acid, exampled by the PIF‐DELLA‐JAZ‐MYC2 regulating module which prioritized defense over growth in Arabidopsis and rice.^[^ ^9^ ^]^ Beyond the above reports, some valuable locus had been reported on positively promoting yield and disease resistance in crop. For example, a natural allele of rice transcription factor *Bsr‐d1* from Digu regulated durable and broad‐spectrum blast disease resistance without yield reduction.^[^ ^10^ ^]^ Rice *IPA1* promoted both yield and disease resistance by sustaining a balance between growth and immunity.^[^ ^11^ ^]^ In wheat, the durable and broad‐spectrum resistance gene with little production penalty was also discovered recently.^[^ ^12^ ^]^ These studies revealed that resistance could be achieved without fitness cost or synergistic style. However, in cotton, upon the most devastating Verticillium wilt (VW), although some resistant genes and their molecular mechanisms were reported, their effects on plant growth and yield had been neglected, which could not supply enough power for breeding.

The previous reports demonstrated that defense signals were mainly perceived at the cell surface and/or in the nucleus, a growing body of evidence indicated that an often‐neglected organelle, chloroplast, also collectively contributed to plant defense responses.^[^ ^13^, ^14^ ^]^ The chloroplast situates in central position in oxygenic photosynthesis and primary metabolism, converting the light into chemical energy and storing it in the form of organic compounds for plant life activities.^[^ ^15^ ^]^ In the process of light conversion to chemical energy, any environmental/pathogenic stimuli to the components of chloroplast may affect the photosynthesis and facilitate the production of by‐products, chloroplast‐derived reactive oxygen species (cROS).^[^ ^16^ ^]^ Correspondingly, chloroplast became the target of pathogens affecting its functions to accelerate colonization in the host.^[^ ^17^ ^]^ The relevant examples provided insight into current state‐of‐knowledge of the chloroplast, however, whether and how crop immunity modulation in chloroplast is influenced by epigenetic regulation such as post‐translational modification (PTM) remains unclear. Especially in cotton, recognizing chloroplast immunity function is still an embryonic field, the chloroplast immunity and its underlying mechanism are still obscure. Our previous finding showed that the photosynthesis was at the top of the significantly enriched metabolic pathways (data not shown) in a widely planted cotton cultivar ND601, with high yield and VW resistance that was scarce in cotton production. Previous report suggested that regulating gene expression and genetic background could help mitigate the balance between immunity and yield in rice.^[^ ^18^ ^]^ This attracts us to unveil the underlying mechanism of chloroplast orchestrating yield and disease resistance in cotton.

Much of present knowledge about the regulation of plant immune responses has been explored through genetic and genomic approaches for the identification of genes and their patterns underlying defense pathways, which were limited to determine the effects of gene loss or gain functions, and powerless to explore dynamic regulation of cellular processes.^[^ ^19^, ^20^ ^]^ Consequently, our current understanding of plant immune networks is far from complete. PTM is a faster and more efficient regulatory system in all layers of plant immune responses to achieve cellular homeostasis.^[^ ^21^ ^]^ Thus, a research shift from studying the underlying transcriptional networks to studying PTM is essential and significant. Here, we firstly identified and characterized the cotton lysine Khib and Ksuc modifications via a large‐scale MS‐based proteomic analysis combining with tandem mass tag (TMT) protein labeling. We discovered that the chloroplast proteins were hit most by the Khib and Ksuc under *Verticillium dahliae* stress, and the both modifications were strongly associated with plant resistance. A chloroplast‐located protein GhPSB27, situating in a core hub both in Khib‐ and Ksuc‐modified proteins network, was identified and promoted plant resistance and biomass‐ and seed‐yield via PTM regulations. Our findings revealed that the PTM mediated chloroplast function orchestrating in plant immunity and production.

## 2 Results

### 2.1 Identification and Characterization of Cotton Khib Sites

**Figure 1:**
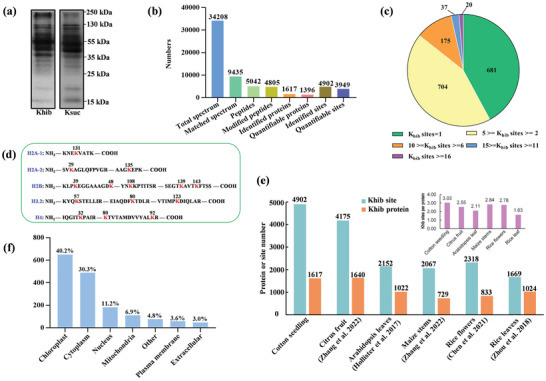
Identification and characterization of the global Khib sites and proteins in cotton. a) Western blotting analysis of Khib and Ksuc with pan‐Khib and Ksuc antibody from resistant cotton cultivar ND601 seedling, respectively. b) Statistical analysis of identified Khib sites, peptides and proteins in resistant cotton cultivar ND601 seedling. c) Distribution of Khib proteins based on the number of Khib sites per identified protein. d) Cotton histone Khib sites identified by mass spectrometry. e) Comparison of Khib sites and proteins among cotton, citrus fruit, Arabidopsis leaves, maize stems, rice flowers and rice leaves. f) Predicted subcellular localization of the Khib proteins.

To study whether lysine acylations exist in cotton, we performed immunoblotting with the pan anti‐Khib and anti‐Ksuc antibodies. Substantial acetylation sites were detected in the proteins extracted from the resistant cotton cultivar ND601 seedlings using the anti‐Khib and anti‐Ksuc antibodies, respectively (**Figure** **1**a). Subsequently, the protein extracts were subjected to a large‐scale MS‐based proteomic analysis and TMT protein labelling to globally identify Khib‐ and Ksuc‐substrate proteins and their modification sites. The three biological replicates were highly correlated, indicating good reproducibility (Figure S1a, Supporting Information). A total of 4902 Khib sites (with a false‐discovery rate (FDR) < 0.01) covering 1617 proteins were identified (Figure 1b; Table S1, Supporting Information). Of the 1617 identified Khib proteins, 42.1% (681/1617) had a single site, 43.5% (704/1617) had two to five sites, and 14.4% (232/1617) contained more than five sites (Figure 1c). The histones H2A, H2B, H3.2 and H4 were modified even by 14 Khib sites (Figure 1d). We discovered more Khib sites and acylated proteins and a higher number of Khib sites per protein in cotton than those in citrus fruit,^[^ ^22^ ^]^ Arabidopsis leaf,^[^ ^23^ ^]^ maize stem,^[^ ^24^ ^]^ and rice leaf and flower (Figure 1e).^[^ ^25^, ^26^ ^]^

To explore the subcellular distribution of Khib proteins, we performed a cellular compartment analysis of the Khib proteins. The results showed that most of the Khib proteins were significantly enriched in the chloroplast (40.2%) and partially in cytoplasm (30.3%) and nucleus (11.2%), indicating that Khib may play important roles in diverse metabolic processes (Figure 1f). On the basis of GO and KEGG analyses, we found that important primary metabolisms were overrepresented, such as carbohydrate, energy and amino acid metabolisms (for example nitrogen metabolism/photosynthesis/arginine biosynthesis/alanine, aspartate and glutamate metabolism/valine, leucine, and isoleucine biosynthesis) (Figure S1b, Supporting Information). Notably, Khib‐modified proteins were also enriched in the GO biosynthetic secondary metabolism, oxidoreductase activity, and response to stimulus (Table S2, Supporting Information). Altogether, these data indicated that the Khib may influence plant stress responses and coordinate cellular metabolic processes.

### 2.2 Detection of Cotton Ksuc and Cross‐Talk Analysis of Khib and Ksuc

**Figure 2:**
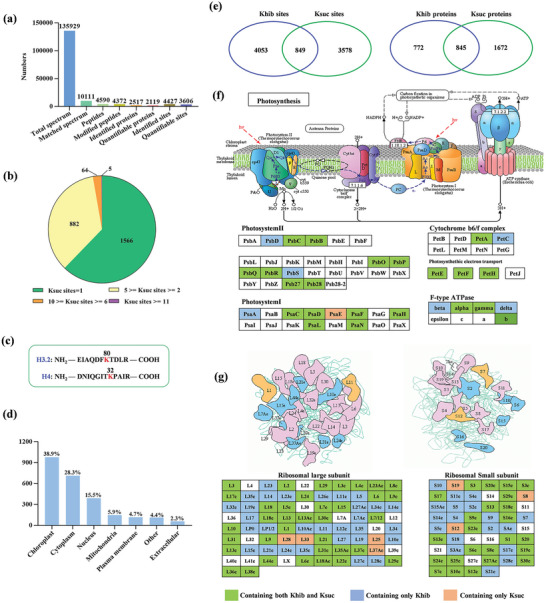
Identification of the global Ksuc proteins in cotton and overlap between Khib and Ksuc of cotton proteins. a) Statistical analysis of identified Ksuc sites, peptides, and proteins in resistant cotton cultivar ND601 seedling. b) Distribution of Ksuc proteins based on the number of Ksuc sites per identified protein. c) Cotton histone Ksuc sites identified by mass spectrometry. d) Predicted subcellular localization of the Ksuc proteins. e) Venn diagram showed the number of sites and proteins with Khib alone, Ksuc alone, or with both modifications. f) Photosynthesis and related pathways and g) ribosomal proteins modified were significantly enriched by either Khib and Ksuc or both, green color indicates protein containing both Khib and Ksuc sites; wathet blue color indicates protein containing only Khib sites; brown color indicates protein containing only Ksuc sites.

We also detected the Ksuc modification in cotton and identified a total of 4427 Ksuc sites located in 2517 proteins (**Figure** **2**a; Figure S2a and Table S3, Supporting Information), with higher protein coverage (2517/4427, 56.9%) than Khib (1617/4902, 33.0%). Out of the 2517 Ksuc proteins, 62.2% (1566/2517) had a single site, 35.0% (882/2517) had 2–5 sites, and 2.8% (232/1617) contained more than 5 sites (Figure 2b). The results indicated that Ksuc proteins contained more single lysine modification site when compared to the Khib. Similar to the Khib, Ksuc modification was also observed in histone H3.2 and H4 (Figure 2c). The subcellular location profiles showed that approximate 82.7% (2081/2517) of Ksuc proteins located in chloroplast, cytoplasm and nucleus (Figure 2d). Subsequent GO and KEGG pathway enrichment analysis showed that the Ksuc proteins were mainly enriched in terms of multiple central metabolic pathways, such as carbon metabolism, carbon fixation in photosynthetic organisms, biosynthesis of amino acids, and glyoxylate and dicarboxylate metabolism (Figure S2, Supporting Information). Enrichment was also identified in aminoacyl‐tRNA biosynthesis and ribosome, suggesting that Ksuc might involve in the regulation of translation and protein biosynthesis. Interestingly, these results of functional enrichment were very similar to those in the Khib, indicating that both PTMs may cooperate in regulating biological processes of cotton.

Interactions between PTMs clearly exist,^[^ ^27^ ^]^ therefore we analyzed the relationship between Khib and Ksuc. We found that 849 sites within 845 proteins were modified both by Khib and Ksuc (Figure 2e), suggesting that the Khib and Ksuc incorporate cross‐talk. We also discovered that almost all major complexes of photosynthetic electron transport systems, such as photosystems (I and II), cytochrome b6f complex, electron transports, and ATP synthases, were modified by both Khib and Ksuc (Figure 2f). Additionally, a higher proportion of the large subunit and small unit of ribosomal proteins were also modified by both Khib and Ksuc (Figure 2g). These results suggest that both Khib and Ksuc proteins were enriched in energy metabolic networks that include photosynthesis, protein synthesis, and degradation.

### 2.3 Khib and Ksuc are Relevant to High Gene Expressions in Response to *V. dahliae*

**Figure 3:**
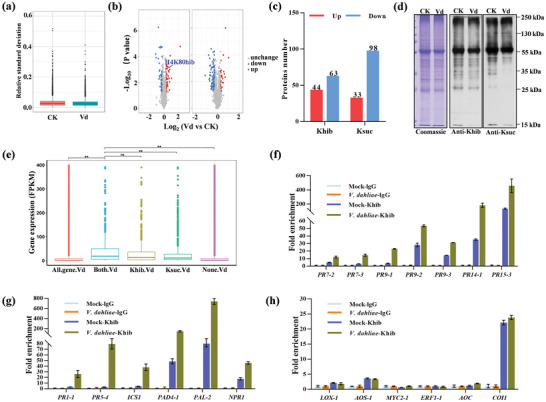
Khib‐ and Ksuc‐modified gene expression and Khib specifically modulate PR and salicylic acid (SA) signaling related gene expression. a) Box plot of relative standard deviation (RSD) distribution of three repeated samples using quantified proteins. CK, water treatment; Vd, *Verticillium dahliae* infection. b) Volcano plot of significantly increased and decreased Khib and Ksuc modification sites in the infected cotton and the CK, respectively. c) The number of differentially expressed Khib and Ksuc proteins in the infected cotton relative to the CK. d) Western blotting analysis of Khib and Ksuc in the infected cotton and the CK seedling. Equal loading was confirmed by Coomassie Brilliant Blue staining (left panel). e) The expression levels of genes that translated proteins contain Khib and/or Ksuc modification sites at 12 h post‐inoculation (hpi) based on transcriptomic data. f–h) chromatin immunoprecipitation‐qPCR (ChIP‐qPCR) analysis of Khib levels at seven PR‐related (f), six SA‐related (g), and six jasmonic acid (JA) related (h) genes in the infected or mock cotton roots, the rabbit lgG was used as negative control. PR, pathogenesis‐related gene; ICS1, isochorismate synthase 1; PAD4, phytoalexin deficient 4; PAL, phenylalanine ammonia‐lyase; NPR1, nonexpressor of pathogenesis‐related genes 1; LOX, lipoxygenase; AOS, allene oxide synthase; MYC2, transcription factor MYC2; AOC, allene oxide cyclase; COI1, coronatine‐insensitive protein 1. Error bars represent the standard deviation (SD) of three biological replicates.

To uncover whether *V. dahliae* infection alters the Khib and Ksuc levels, we detected the Khib and Ksuc levels and discovered modified difference after *V. dahliae* infection (Tables S4 and S5, Supporting Information). To avoid the possibility that the changes in Khib or Ksuc might be caused by the alteration in protein abundance, we standardized Khib and Ksuc proteome data with corresponding proteome under three high‐correlated biological replicates (**Figure** **3**a). Bioinformatics analysis showed that *V. dahliae* infection resulted in the changes of Khib and Ksuc levels compared to the uninfected (CK) (Figure 3b,c), further the changes were confirmed by WB (Figure 3d). To unveil the potential roles of Khib and Ksuc in regulating gene expression, we analyzed the expression level of genes which encoded proteins with Khib and/or Ksuc modifications under *V. dahliae* stress. The genes encoding proteins with Khib modification had higher expression level than those with Ksuc, and the genes with both PTMs showed higher expression than those with Ksuc alone rather than Khib (Figure 3e). These results suggested that the enrichments of Khib and Ksuc were correlated with high gene expression, especially of Khib.

Among the changed modification level proteins, we found a histone H4K80hib significantly increased upon *V. dahliae* stress (Figure 3b). Previous studies in animal cells proved that histone lysine acylations markedly affected gene expression,^[^ ^28^ ^]^ while it was seldom studied in plants. We further performed anti‐Khib chromatin immunoprecipitation‐qPCR (ChIP‐qPCR) assay to amplify the promoter regions of pathogenic related protein (PR) and defense‐related genes in SA and JA signaling pathways (Figure S3, Supporting Information), and found that the Khib level in the infected cotton significantly increased at the promoter of seven PR and six SA‐dependent genes compared to the noninfected cotton (Figure 3f–i), whereas, the genes in the JA signaling pathway were not detected at all (Figure 3j,k). These indicated that the Khib‐modified proteins specifically modulate defense‐related gene expression during *V. dahliae* infection.

### 2.4 Khib and Ksuc are Strongly Associated with Plant Resistance to *V. dahliae*

**Figure 4:**
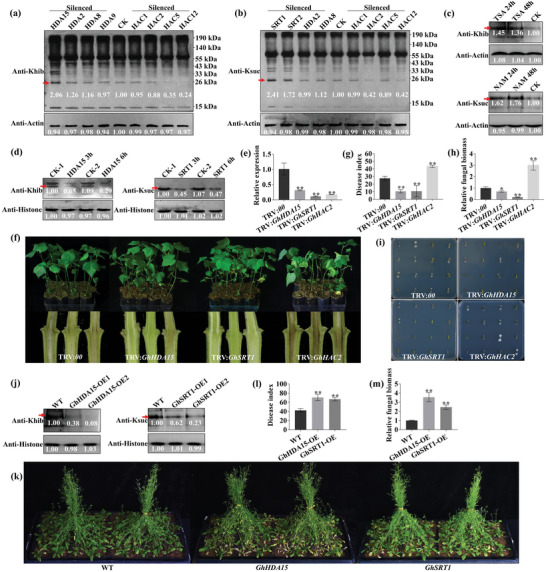
Identification and functional study of writer and eraser for Khib and Ksuc in plants. a) Khib levels analysis in the silenced and CK cottons by immunoblotting. Anti‐Actin antibody serves as loading control. b) Ksuc levels analysis in the silenced and CK cottons by immunoblotting. Anti‐Actin antibody serves as loading control. c) Change of Khib and Ksuc levels in the cotton leaves at 24 and 48 h treated with trichostatin A (TSA) and nicotinamide (NAM), respectively. Anti‐Actin antibody serves as loading control. d) Eraser function validation of GhHDA15 and GhSRT1 in vitro. The purified proteins were coincubated with cotton histones for 3 and 6 h and determined by immunoblotting analysis. Anti‐Histone H3 antibody serves as loading control. e) Detection of silencing efficiency for GhHDA15 (TRV:GhHDA15), GhSRT1 (TRV:GhSRT1) and GhHAC2 (TRV:GhHAC2) in cottons by qRT‐PCR. The empty vector (TRV:*00*) plants served as control. f) Verticillium wilt (VW) symptoms and stem vascular discoloration symptoms at 25 dpi. g) Disease index (DI) of seedlings at 25 dpi. h) *Verticillium dahliae* isolation from the infected stems at 10 dpi. Colonies of *V. dahliae* were observed after three days of culture. i) Detection of fungal biomass from the first true leaves by qRT‐PCR. j) Khib and Ksuc levels analysis in the transgenic Arabidopsis by immunoblotting. Anti‐Histone H3 antibody serves as loading control. k) VW symptoms of wild‐type (WT), the transgenic Arabidopsis of GhHDA15 and GhSRT1 at 25 dpi. l) DI at 25 dpi. m) Biomass of *V. dahliae* from the infected seedlings was quantified by qRT‐PCR. Values are means with standard deviation (SD) (*n* = 3 biological replicates). Error bars represent the SD of three biological replicates. Asterisks indicate statistically significant differences according to Student's *t*‐test (two‐tailed) (^*^ *p* < 0.05; ^**^ *p* < 0.01). All experiments were repeated at least three times.

Mammalian sirtuin and Zn‐finger HDAC proteins were reported to have debutyrylase and/or decrotonylase activities.^[^ ^29^ ^]^ To test whether the similar sirtuin and HDAC proteins existed in cotton, we silenced *SRT*, *HDA*, and *HAC* homologous genes (*GhSRT1*, *GhSRT2*; *GhHDA15*, *GhHDA2*, *GhHDA8*, *GhHDA9*; *GhHAC1*, *GhHAC2*, *GhHAC5*, and *GhHAC12*) in cotton (Figure S4, Supporting Information), and determined the change of modification level by immunoblotting with the pan‐anti‐Khib and pan‐anti‐Ksuc antibody, respectively. An obvious increase in Khib band intensity was observed in the *GhHDA15* and *GhHDA2* knock‐down plants, and an obvious decrease in *GhHAC5* and *GhHAC12* knock‐down plants (**Figure** **4**a). For Ksuc, an obvious increase band intensity was observed in *GhSRT1* and *GhSRT2* knock‐down plants, and an obvious decrease in Ksuc level in *GhHAC2* and *GhHAC12* knock‐down plants (Figure 4b). Considering that trichostatin A (TSA) and nicotinamide (NAM) is the inhibitor of HDA and SRT family deacetylases, respectively,^[^ ^30^, ^31^ ^]^ we treated cotton seedlings using TSA and NAM to determine whether cotton Khib and Ksuc could be regulated by HDA and SRT, respectively. Consistently, the Khib level in the TSA‐treated plants and the Ksuc level in the NAM‐treated plants significantly increased compared with the CK plants, and the Ksuc level increased more after NAM treatment (Figure 4c). To further validate these results, we expressed GhHDA15, GhSRT1, and GhHAC12 in *Escherichia coli* to obtain purified GhHDA15, GhSRT1, and GhHAC12 proteins, respectively. The cotton total histones were used as substrate. As expected, in vitro enzyme activity assay, the Khib and Ksuc levels of the histones were decreased during GhHDA15 and GhSRT1 protein treatment, respectively (Figure 4d). Unfortunately, GhHAC12 prokaryotic expression protein was not purified due to its high molecular weight. Collectively, the above results suggest that GhHDA15 and GhSRT1 have deacylase activity to erase Khib and Ksuc in cotton, respectively, meanwhile, GhHAC2, GhHAC5, and GhHAC12 have acetyltransferase activity to write Khib and Ksuc.

To validate whether the altering Khib and Ksuc levels affected plant VW resistance, we identified the resistance level for the *GhHDA15*, *GhSRT1*, and *GhHAC2* silenced cottons (Figure 4e). At 25 days post‐inoculation (dpi), the silenced *GhHDA15* (tobacco rattle virus (TRV):*GhHDA15*) and *GhSRT1* (TRV:*GhSRT1*) cottons which resulted in increased Khib and Ksuc levels, respectively, showed enhanced VW resistance, making the disease reaction type from resistant (R, 10 < DI ≤ 20) to high resistant (HR, 0 < DI ≤ 10). Conversely, the silenced *GhHAC2* (TRV:*GhHAC2*) cottons was more susceptible than the TRV:*00* (CK) (Figure 4f,g). The vascular bundles and fungal restore assay of two deacetylase genes and TRV:*GhHAC2* cottons also validated the results (Figure 4h,i). We further overexpressed *GhHDA15*, *GhSRT1* in Arabidopsis and found that the Khib and Ksuc levels significantly decreased in the transgenic lines, respectively (Figure 4j). Moreover, the transgenic plants displayed more susceptible (Figure 4k,l) and higher *V. dahliae* biomass (Figure 4m) than the wild‐type (WT). To exclude whether Kac and Kcr were regulated by *GhHDA15* and *GhSRT1*, we performed immunoblotting with the pan anti‐Kac/Kcr antibodies and discovered that the Kac and Kcr levels in silenced GhHDA15 and GhSRT1 cottons were similar to CK (Figure S5, Supporting Information), indicating that Kac and Kcr were not regulated by GhHDA15 and GhSRT1. These results suggested that Khib and Ksuc regulated by GhHDA15, GhSRT1, and GhHAC2 were strongly associated with plant resistance to *V. dahliae*.

### 2.5 Chloroplast Proteins are Hit most During Khib and Ksuc Modifications

**Figure 5:**
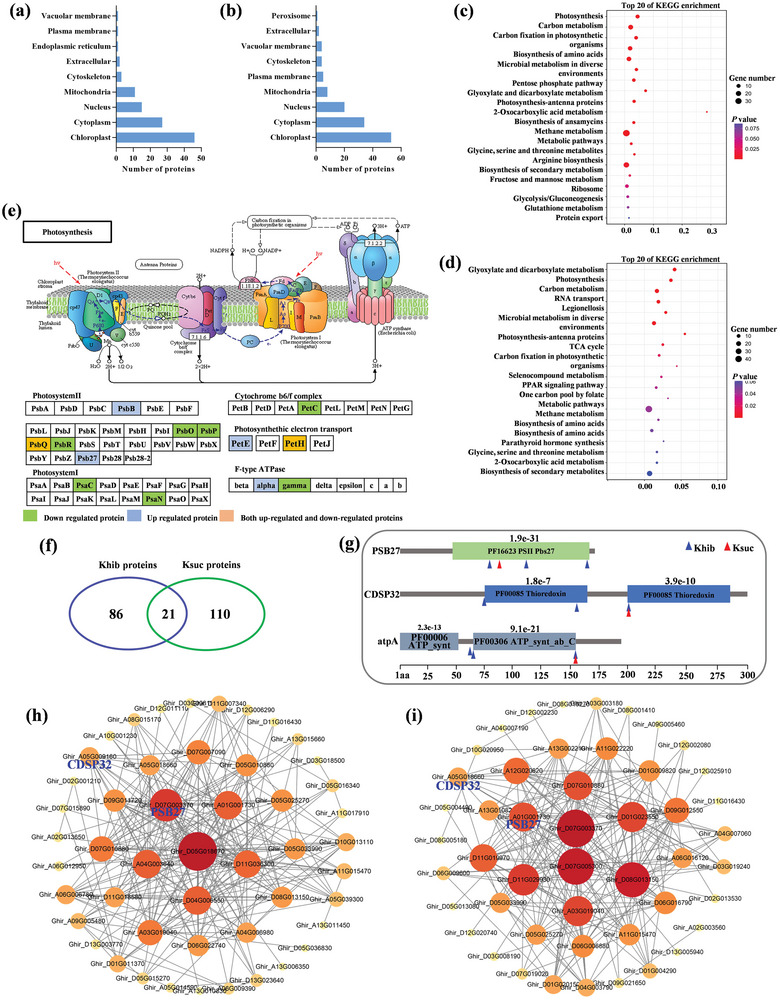
Chloroplast proteins are most enriched in differentially regulated Khib‐ and Ksuc‐proteins upon *Verticillium dahliae* infection. a,b) Predicted subcellular localization of the differentially Khib‐ and Ksuc‐modified proteins in cotton. c,d) KEGG analysis of the differently Khib‐ and Ksuc‐modified proteins in cotton. e) Photosynthesis and related pathways were significantly enriched by either differentially Khib‐ and Ksuc‐modified proteins or both. Green color indicates down‐regulated protein; wathet blue color indicates upregulated protein; brown color indicates both down‐ and upregulated proteins. f) Overlapping proteins with the differentially Khib/Ksuc‐modified after *V. dahliae* infection. g) Peptide domain prediction in GhPSB27, GhCDSP32, and GhCAP10A. *E*‐value represents the confidence of the predicted domains; the blue and red triangles represent differentially Khib‐ and Ksuc‐modified sites, respectively. h) Protein–protein interaction network for differentially Khib‐modified proteins. i) Protein–protein interaction network for differentially Ksuc‐modified proteins.

Based on the Khib and Ksuc cross‐talk analysis, we found that a large proportion of differential Khib and Ksuc‐modified proteins (43.0% and 40.5%, respectively) were located in the chloroplast (**Figure** **5**a,b). KEGG analysis showed that the Khib‐ and Ksuc‐modified proteins were enriched in several pathways related to energy metabolism, including photosynthesis, carbon metabolism, glyoxylate and dicarboxylate metabolism, and photosynthesis‐antenna proteins. (Figure 5c,d). Among them, a large proportion of the differential Khib‐ and Ksuc‐modified proteins belonged to photosynthetic electron transport systems, such as photosystems (I and II), cytochrome b6f complex, electron transports, and ATP synthases (Figure 5e). Of the 21 differentially modified proteins harboring both the Khib and Ksuc sites (Figure 5f), 11 were chloroplast proteins, including eight with a Khib site and a Ksuc site (Table S6, Supporting Information). The other three proteins GhPSB27, GhCDSP32, and GhCAP10A contained three Khib sites and one Ksuc site (Figure 5g). Specifically, for GhPSB27, a famous protein functioning in photosynthetic processes, all four sites were situated in the conserved domains. We also found that the majority of differentially modified proteins formed a highly interconnected protein‐protein interaction network (Figure 5h,i), and the GhPSB27, GhCDSP32 located at the important node, especially GhPSB27 in a near core hub, suggesting the important regulatory function of GhPSB27 on VW resistance and/or growth.

### 2.6 Acylated Photosystem II Repair Protein GhPSB27 Significantly Enhances Plant VW Resistance

**Figure 6:**
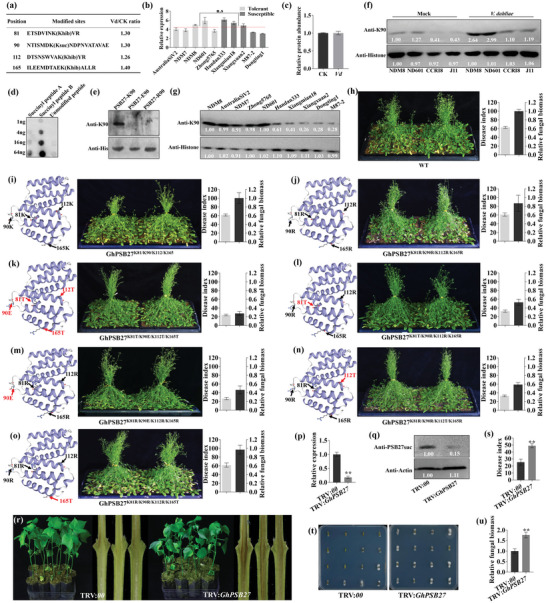
GhPSB27 regulates Verticillium wilt (VW) resistance via Khib and Ksuc. a) The modified sites information of GhPSB27. b) *GhPSB27* expression in five resistant and five susceptible cotton cultivars by qRT‐PCR analysis. c) Relative protein abundance of GhPSB27 in CK and infected resistant cultivar ND601. d) Dot blot assay of the specific GhPSB27^K90suc^ antibody, the dot‐blot assays were carried out using GhPSB27^K90suc^ antibody (1:1000). e) GhPSB27 K90 mutation recombinant proteins were detected using sequence‐specific anti‐GhPSB27^K90suc^ antibody. Anti‐His tag antibody serves as loading control, the specific K90 antibody can exclusively recognize the GhPSB27K90suc recombinant protein, but not its mutant counterpart GhPSB27^E90^ and GhPSB27^R90^. f) GhPSB27^K90suc^ levels in two resistant (NDM8 and ND601) and two susceptible (CCRI8 and J11) cotton cultivars at 12 hpi. Anti‐Histone H3 antibody serves as loading control. g) The modified level of GhPSB27^K90suc^ in five resistant and five susceptible cotton cultivars. Anti‐Histone H3 antibody serves as loading control. h) VW symptom, disease index (DI), and fungal biomass of wild‐type (WT) Arabidopsis. i–o) Three‐dimensional structure, VW symptom, DI, and fungal biomass of normal GhPSB27, site‐directed mutants GhPSB27^K81R/K90R/K112R/K165R^, GhPSB27^K81T/K90E/K112T/K165T^, GhPSB27^K81T/K90R/K112R/K165R^, GhPSB27^K81R/K90E/K112R/K165R^, GhPSB27^K81R/K90R/K112T/K165R^, and GhPSB27^K81R/K90R/K112R/K165T^. p) Detection of silencing efficiency for *GhPSB27* (TRV:*GhPSB27*) in cottons by qRT‐PCR. q) The modified level of GhPSB27^K90suc^ in the TRV:*GhPSB27* and empty vector (TRV:*00*) cottons. Anti‐Actin antibody serves as loading control. r–u) VW symptoms, stem vascular discoloration symptoms, DI, *Verticillium dahliae* isolation, and relative fungal biomass of the TRV:*GhPSB27* and TRV:*00* cottons. Values are means with standard deviation (SD) (*n* = 3 biological replicates). Error bars represent the SD of three biological replicates. Asterisks indicate statistically significant differences according to Student's *t*‐test (two‐tailed) (^**^ *p* < 0.01). All experiments were repeated at least three times.

After *V. dahliae* inoculation, we found that the modified levels of four sites in the GhPSB27 were increased (**Figure** **6**a), however, *GhPSB27* transcript abundance was similar between the resistant and the susceptible cottons after *V. dahliae* infection (Figure 6b), and the relative protein abundance of GhPSB27 in CK and infected resistant cultivar ND601 was also similar (Figure 6c), suggesting that *GhPSB27* possibly involved in VW resistance in PTM level rather than the transcriptional level. We explored the GhPSB27^K90suc^ specific antibody in the K90 site to validate the change of GhPSB27 modification level, the GhPSB27^K90suc^ antibodies showed good specificities, as shown by dot‐blot and K90 point mutant assays (Figure 6d,e). The result showed that the GhPSB27^K90suc^ level was significantly increased upon *V. dahliae* infection, especially in the resistant cultivars (Figure 6f). And the GhPSB27^K90suc^ levels were strongly associated with VW resistance, as reflected in five resistant‐ and susceptible‐cottons (Figure 6g).

To determine whether the four Khib and Ksuc sites in GhPSB27 participated in VW resistance, we performed the site‐directed mutants on target sites, GhPSB27^K81^, GhPSB27^K90^, GhPSB27^K112^, and GhPSB27^K165^, individually. The lysine modification sites were mutated to threonine (T), glutamic acid (E), and arginine (R) to mimic Khib, Ksuc and unmodified, respectively.^[^ ^32^, ^33^, ^34^ ^]^ Subsequently, we developed overexpressing Arabidopsis with a normal GhPSB27 (GhPSB27^K81/K90/K112/K165^) and six site‐directed mutants (GhPSB27^K81R/K90R/K112R/K165R^, GhPSB27^K81T/K90E/K112T/K165T^, GhPSB27^K81T/K90R/K112R/K165R^, GhPSB27^K81R/K90E/K112R/K165R^, GhPSB27^K81R/K90R/K112T/K165R^, and GhPSB27^K81R/K90R/K112R/K165T^) (Figure S6, Supporting Information). The six mutated GhPSB27 protein structures were almost the same as the normal GhPSB27 (Figure 6h–o). After inoculation, the mutants GhPSB27^K81T/K90E/K112T/K165T^, GhPSB27^K81T/K90R/K112R/K165R^, GhPSB27^K81R/K90E/K112R/K165R^, and GhPSB27^K81R/K90R/K112T/K165R^ exhibited enhanced VW resistance, decreased disease indexes (DIs), and less fungal biomass compared to the WT, especially the mutant GhPSB27^K81T/K90E/K112T/K165T^ (Figure 6h, k–n). In contrast, the normal GhPSB27, mutant GhPSB27^K81R/K90R/K112R/K165T^, and unmodified mutant GhPSB27^K81R/K90R/K112R/K165R^ did not display significant difference (Figure 6h–j,o).

We further employed VIGS strategy to knock‐down the expression of *GhPSB27* (Figure 6p) and found that the GhPSB27^K90suc^ level was significantly decreased compared to the TRV:*00* (CK) plants (Figure 6q). On the condition of *V. dahliae* infection, the silenced cottons displayed more severe VW symptoms at 25 dpi, more obvious brown changes of vascular bundles, higher DI (49.1), and more fungal amounts than the CK plants (Figure 6r–u). Together, these results indicated that GhPSB27 positively regulated cotton VW resistance via Khib and Ksuc modifications.

### 2.7 GhPSB27 Modifications Result in Plant Biomass‐ and Seed‐Yield Increment

**Figure 7:**
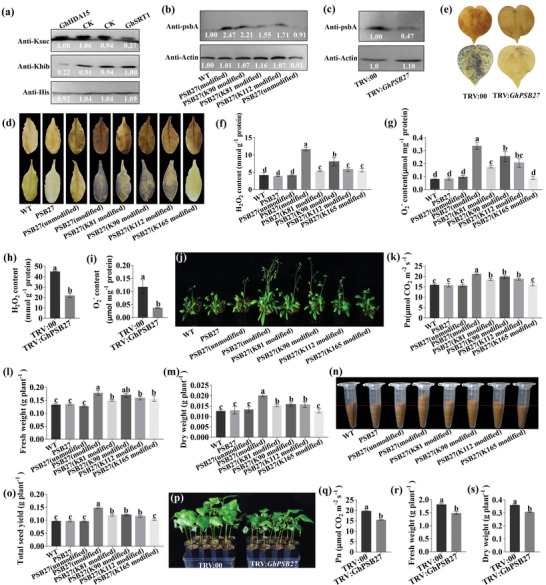
Acylated GhPSB27 enhances plant disease resistance and increases yield via regulating D1 protein content. a) Eraser function validation of GhHDA15 and GhSRT1 in vitro. The purified GhHDA15 and GhSRT1 proteins were coincubated with GhPSB27 proteins for 3 h and determined by immunoblotting analysis. Anti‐His tag antibody serves as loading control. b) D1 protein contents were determined in the five site‐directed mutants Arabidopsis with D1 protein (PsbA) C‐terminal specific antibody by Immunoblotting. PSB27^(modified)^, PSB27^(K81 modified)^, PSB27^(K90 modified)^, PSB27^(K112 modified)^, PSB27^(unmodified)^ represent GhPSB27^K81T/K90E/K112T/K165T^, GhPSB27^K81T/K90R/K112R/K165R^, GhPSB27^K81R/K90E/K112R/K165R^, GhPSB27^K81R/K90R/K112T/K165R^, represent GhPSB27^K81R/K90R/K112R/K165R^. Anti‐Actin antibody serves as loading control. c) The D1 protein contents of the CK (TRV:*00*) and *GhPSB27* (TRV:*GhPSB27*) cottons were analyzed by immunoblotting. Anti‐Actin antibody serves as loading control. d,e) DAB staining of H_2_O_2_ and NBT staining of O_2_^−^ in silenced cotton and transgenic Arabidopsis. f,g) Determination of H_2_O_2_ and O_2_^−^ contents in transgenic Arabidopsis. PSB27^(K165 modified)^ represent GhPSB27^K81R/K90R/K112R/K165T^. h,i) Determination of H_2_O_2_ and O_2_^−^ contents in silenced cotton. j) Growth vigor of four‐week‐old transgenic Arabidopsis. k–m) Net photosynthetic rate, fresh weight and dry weight of the wild‐type (WT), and transgenic Arabidopsis. n) The amounts of mature seeds per five plants from WT and transgenic lines. o) Total seed yield of per plant for WT and transgenic lines. p) Growth vigor of three‐week‐old TRV:*00* and TRV:*GhPSB27* cottons. q–s) Net photosynthetic rate, fresh weight, and dry weight of 3‐week‐old TRV:*00* and TRV:*GhPSB27* cottons. Values are means with standard deviation (SD) (*n* = 3 biological replicates). Error bars represent the SD of three biological replicates, Lowercase letters represent statistically significant differences (*p* < 0.05) according to Tukey's honest significant difference (HSD) test. All experiments were repeated at least three times.

We further verified GhHDA15 and GhSRT1 could remove the Khib and Ksuc of GhPSB27 by in vitro experiments with purified GhPSB27, respectively (**Figure** **7**a). GhPSB27 is a small thylakoid lumen‐localized protein known to serve as an assembly factor for the photosystem (PS) II core proteins D1.^[^ ^35^ ^]^ We speculated that the modification levels of GhPSB27 might be involved in the D1 protein synthesis. To test this hypothesis, we detected D1 protein content with a PsbA C‐terminal specific antibody and found that the D1 protein content of four resistant mutant lines GhPSB27^K81T/K90E/K112T/K165T^, GhPSB27^K81T/K90R/K112R/K165R^, GhPSB27^K81R/K90E/K112R/K165R^, and GhPSB27^K81R/K90R/K112T/K165R^ were significantly increased compared to the normal GhPSB27, unmodified mutant GhPSB27^K81R/K90R/K112R/K165R^, and WT (Figure 7b). Inversely, the D1 protein content was significantly decreased in the silenced cottons (Figure 7c). Considering the function of D1 protein in promoting ROS production in chloroplasts, we further detected the hydrogen peroxide (H_2_O_2_) and superoxide anion (O_2_^−^) and found that higher H_2_O_2_ and O_2_^−^ in the four resistant mutant lines and lower in the silenced cottons (Figure 7d–i). Taken together, these results indicated that modified GhPSB27 played an important role in ROS accumulation in plants.

Given that the D1 protein plays a central role in the PSII, we infer that the change of D1 protein content caused by GhPSB27 modification will affect photosynthesis and yield traits in plants. We observed that the four resistant mutants with high D1 protein content displayed more vigorous growth, much more and larger rosette leaves (Figure 7j), higher photosynthetic physiological indexes (Figure 7k; Figure S7a–d, Supporting Information), especially the mutant K81T/K90E/K112T/K165T. Correspondingly, the fresh and dry weight of these mutant lines also showed the same trend (Figure 7l,m). The stronger photosynthetic rate and the better growth potential eventually caused a greatly increased seed yield in the four mutant lines (Figure 7n,o). Inversely, the reduction of D1 protein caused by GhPSB27 modification in silenced cottons resulted in a significant reduction in growth potential (Figure 7p), photosynthetic rate, SPAD value (Figure 7q; Figure S7e–h, Supporting Information), and dry and fresh weight (Figure 7r,s). Taken together, these results indicated that GhPSB27 regulated D1 protein content via Khib and Ksuc, thereby improving both VW resistance and yield in plants.

## 3 Discussion

VW threatens the cotton production worldwide, and serious outbreaks can remarkably reduce fiber yield.^[^ ^36^ ^]^ Scientists have been trying to mitigate the negative effects of stress on cotton productivity though challenged. An ideal breeding objective is to develop new cultivar with high yield and immunity to pathogens. Frequently, however, enhanced defense is achieved at the cost of other processes, coming with yield penalties.^[^ ^37^ ^]^ Of which, a versatile regulatory pattern rapidly shifting plant energy is essential. Till now, much of knowledge about the regulation of plant immune responses has been discovered via genetic and genomic approaches.^[^ ^10^, ^38^ ^]^ In cotton, on the one hand, a number of genes and the underlying mechanism involved in VW resistance have been exposed based on reverse genetics, such as cell wall modifications,^[^ ^39^ ^]^ extracellular enzymes,^[^ ^40^ ^]^ pattern recognition receptors,^[^ ^41^ ^]^ transcription factors,^[^ ^42^ ^]^ and SA/JA/ET‐related signal transduction pathways.^[^ ^43^, ^44^ ^]^ On the other hand, several genetic loci were revealed responsible to VW resistance based on GWAS and QTLs mapping.^[^ ^10^, ^45^, ^46^ ^]^ The aforementioned reports gradually build up our understanding to cotton defense to *V. dahliae* mainly dependent on root tissue. However, the current knowledge supplied more information on how cotton defends against pathogen stress, whereas much less is known about how defense signaling modulates host growth, and vice versa. Of the most important organelles, the chloroplast has recently emerged to coordinate plant defense responses,^[^ ^17^ ^]^ however, the molecular mechanism that crop immunity balances with yield modulated by chloroplast is unclear.

In the present study, we reveal a mechanism balancing cotton VW resistance and yield through Khib and Ksuc modifications of chloroplast protein. We firstly identified Khib and Ksuc sites across cotton genome under *V. dahliae* stress, and discovered that both modifications were strongly associated with VW resistance. Of which, the chloroplast proteins were hit most, indicating that the potential importance of Khib and Ksuc in chloroplast immunity. Further KEGG analysis highlighted that photosynthetic electron transport and ribosomal subunits were susceptible to both Khib and Ksuc modifications. To our knowledge, photosynthesis in plant chloroplast transforms light energy into chemical energy to provide organic nutrients, which has become an essential chemical reaction for the survival of green plants.^[^ ^47^ ^]^ Ribosomes are macromolecular machines, existing within all living cells, that perform biological protein synthesis (mRNA translation).^[^ ^48^ ^]^ These supply an important clue that the chloroplast is very likely to regulate plant growth and resistance via Khib and Ksuc modifications, as reflected that reaching to 43.0% and 40.5% for differential Khib and Ksuc‐modified proteins located in chloroplast. We evidenced that changing the modification level of Khib and Ksuc vividly altered cotton VW resistance, however, the cotton key elements that regulate this post‐translational lysine acylation pathway remain unclear. We firstly identified cotton GhHDA15, GhSRT1, and GhHAC2 as the enzymes to remove and add Khib and Ksuc, respectively, regarding as the “eraser” and “writer” for Khib‐ and Ksuc‐substrates. And the preferentially expressed GhHDA15 relative to GhSRT1 and GhHAC2 post‐inoculation indicated its predominant role in Khib modification. Interestingly, the *GhHDA15* expression was induced by *V. dahliae* only in susceptible cotton cultivars, but not in resistant cottons (Figure S8, Supporting Information), which should lead to a higher Khib level in resistant cottons than the susceptible. This was consistent with the above result that knock‐down of *GhHDA15* resulted in enhanced VW resistance in cotton.

In photosynthetic organisms, PSB27 protein is one of the important assembly and repair factors, which plays an important role in maintaining the efficient assembly and repairs of D1 protein that functions a central role in photosystem II (PSII) under stress conditions.^[^ ^49^ ^]^ Thus, it is important to maintain and/or increase the rate of its synthesis to improve the repair efficiency of PSII under pathogen stress. Here, we demonstrated that GhPSB27 keeping higher Khib and Ksuc level could promote to repair D1 protein and increase photosynthesis as well as by‐products of cROS, leading to improved plant productivity and disease resistance. In details, the transgenic Arabidopsis of artificially modified PSB27 with more Khib and Ksuc levels (GhPSB27^K81T/K90E/K112T/K165T^) displayed better VW resistance and higher plant yield than other mutants, vividly reflecting the power of site‐directed modification in regulating PSB27 function. We also found that transforming the normal GhPSB27 from ND601 into Arabidopsis could not significantly improve VW resistance of transgenic plants, inferring that the Khib/Ksuc levels of GhPSB27 could not be maintained under Arabidopsis background, leading to impaired function of GhPSB27. Noteworthily, the increase of crop yield was not closely associated with photosynthesis rate based on reported field experiments, such as in wheat, rice, and soybean.^[^ ^50^, ^51^, ^52^ ^]^ Considering that crop yield was a multifaceted outcome of many resources and processes,^[^ ^53^ ^]^ the above point from all grain crops probably is worth thinking over. It was proved that the availability and acquisition of nutrients and water were defined as the limits of crop grain yields.^[^ ^54^ ^]^ In our present study, a novel aspect of epigenetic regulation of chloroplast protein GhPSB27 could simultaneously promote yield and disease resistance, which is interesting and arrestive. Cotton has evolved to be a drought‐resistant crop and can maintain high yield in relatively barren fields, far different from the grain crops that require abundant nutrient and water acquisition.^[^ ^55^ ^]^ Combining these diverse physiology characteristics, it is more likely to accept our current result that photosynthesis is a crucial factor affecting cotton yield. Moreover, maintaining relative high photosynthesis under *V. dahliae* stress should be more important for stable yield.

**Figure 8:**
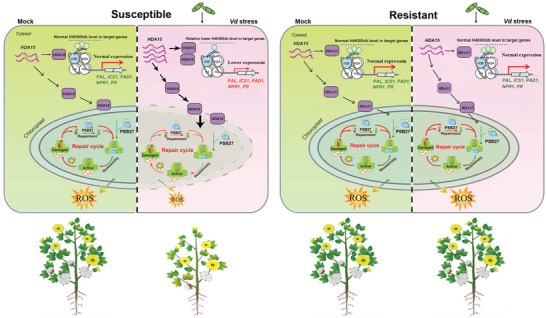
A work model for the modified GhPSB27^Khib^. The *GhHDA15* expression is induced by *Verticillium dahliae* specifically in susceptible cottons, which results in relatively high Khib removing activity and plant susceptibility. In contrast, the resistant cotton can maintain Khib at relatively high level, and specifically dynamically upregulates defense genes involving in salicylic acid (SA) pathway. At the same time, highly modified GhPSB27^Khib^ can increase the contents of D1 protein during the PSII repair cycles and thus increase the contents of chloroplast‐derived reactive oxygen species (cROS) and photosynthesis efficiency of the plants, the two aspects contribute to the enhancement of plant disease resistance and yield.

Finally, we highlight our findings in a work model as illustrated in **Figure** **8**. *Verticillium dahliae* could induce high expression of *GhHDA15* specifically in susceptible cottons, resulting in higher Khib removing activity from its substrate GhPSB27. In contrast, relative high level of GhPSB27^Khib^ in the resistant cottons promoted D1 protein repair, making plants keep higher photosynthetic efficiency and cROS; at the same time, the enhanced lysine acylation by Khib in H4K80hib under *V. dahliae* specifically dynamically upregulated defense genes. The two aspects contribute to plant yield and disease resistance increasing. We noticed that Khib/Ksuc level of GhPSB27 significantly increased under *V. dahliae* stress, and maintained vividly higher epigenetic modification levels in resistant cottons than susceptible, suggesting that there maybe have other undiscovered modulator functioning as the “writer”, together with GhHAD15 to coregulate GhPSB27. These findings have supplied an immediate and feasible resolution to boost plant biomass and crop productivity under pathogen stress, which should have the potential contribution to engineering stress‐resistant and high‐yielding crops.

## 4 Experimental Section

### Plant Materials and *V. dahliae* Strain

The resistant cotton cultivar ND601 exhibiting high yield and VW resistance was bred by the laboratory and planted in a vast area. Five VW tolerant and five VW susceptible cultivars were selected from a natural population comprising 1081 *G. hirsutum* accessions,^[^ ^20^ ^]^ and the detailed information of ten cotton cultivars were presented in the previous study.^[^ ^56^ ^]^ The cotton seedlings were grown in 50% Hoagland's solution under greenhouse with a 16 h light/8 h dark photoperiod and 26 °C (day)/23 °C (night) temperatures for 3 weeks. Subsequently, the cotton seedlings were removed into either a conidial suspension of *V. dahliae* or sterile water (for the control). The seedlings were harvested at 12 h post‐inoculation (hpi), quickly frozen in liquid nitrogen and stored at −80 °C. The aggressive defoliating *V. dahliae* strain LX2‐1 was used in this study. The preparation of conidial suspensions was performed as described previously.^[^ ^57^ ^]^

### Protein Extraction, Trypsin Digestion, and TMT Labeling

Sample was first grinded in liquid nitrogen, then the powder was transferred to 5 mL centrifuge tube and sonicated three times on ice in lysis buffer (including 1% TritonX 100, 10 × 10^−3^ m dithiothreitol, and 1% Protease Inhibitor Cocktail, 50 × 10^−6^ m PR‐619, 3 × 10^−6^ m TSA, 50 × 10^−3^ m NAM, and 2 × 10^−3^ m EDTA). An equal volume of Tris‐saturated phenol was added. After centrifugation at 4 °C, the upper phenol phase was transferred to a new centrifuge tube. Proteins were precipitated by adding at least four volumes of ammonium sulfate saturated methanol and incubated at −20 °C for at least 6 h. After centrifugation at 4 °C, the remaining precipitate was washed with ice cold methanol once, followed by ice cold acetone for three times. The protein was redissolved in 8 m urea and the protein concentration was determined with BCA kit according to the manufacturer's instructions.

For digestion, the protein solution was reduced with 5 × 10^−3^ m dithiothreitol for 30 min at 56 °C and alkylated with 11 × 10^−3^ m iodoacetamide for 15 min at room temperature in darkness. The protein sample was then diluted by adding 100 × 10^−3^ m TEAB to urea concentration less than 2 m. Finally, trypsin was added at 1:50 trypsin to protein mass ratio for the first digestion overnight and 1:100 trypsin to protein mass ratio for a second 4 h digestion. After trypsin digestion, peptide was desalted by Strata X C18 SPE column (Phenomenex) and vacuum dried. Peptide was reconstituted in 0.5 m TEAB and processed according to the manufacturer's protocol for TMT kit.

### Protein Khib and Ksuc Peptides Enrichment and HPLC−MS/MS Analysis

To enrich Khib‐ and Ksuc‐modified peptides, tryptic peptides dissolved in NETN buffer (100 × 10^−3^ m NaCl, 1 × 10^−3^ m EDTA, 50 × 10^−3^ m Tris‐HCl, 0.5% NP‐40, pH 8.0) were incubated with pre‐washed pan antibody beads (PTM‐804 and PTM‐402, respectively. PTM Bio) at 4 °C overnight with gently shaking. With the immobilization of highly specific anti‐Khib and Ksuc antibody, the antibody beaded agarose selectively captures peptides/proteins bearing 2‐hydroxyisobutyryllysine and succinyllysine residues, respectively, but does not cross‐react with the peptides/proteins bearing other structurally similar modified residues. The beads were washed four times with NETN buffer and twice with ddH_2_O after the incubation. The bound peptides were eluted from the beads with 0.1% trifluoroacetic acid and vacuum dried. The LC–MS/MS analysis was performed as previously described with a few changes.^[^ ^25^ ^]^

### Database Search

The resulting MS/MS data were processed using Maxquant search engine (v.1.5.2.8). Tandem mass spectra were searched against *G. hirsutum* database (70 199 sequences) concatenated with reverse decoy database.^[^ ^58^ ^]^ Trypsin/P was specified as cleavage enzyme allowing up to four missing cleavages. The mass tolerance for precursor ions was set as 20 ppm in First search and 5 ppm in Main search, and the mass tolerance for fragment ions was set as 0.02 Da. Carbamidomethyl on cysteine was specified as fixed modification. Khib or Ksuc modification and oxidation on methionine were specified as variable modifications. FDR was adjusted to a quantity of 1% and minimum score for modified peptides was set >40.

### Differentially Modified Lysine Khib and Ksuc Analysis

To reduce impact of changes in protein abundance on the modification, modification quantification was divided by the protein quantification after averaging three replicates. According to the quantification analysis, adjusted *p* value < 0.05 and 1.2‐fold change was set, the ratio greater than 1.2 was defined as upregulated. Similarly, the ratio less than 1/1.2 was defined as down‐regulated.

### VIGS Assay

The conserved nucleotide acid sequences were separately cloned and integrated into TRV binary vector pTRV2. The endogenous cloroplastos alterados 1 gene (*CLA1*) was used to evaluate the effectiveness of VIGS. The VIGS assay was performed according to what previously described.^[^ ^59^ ^]^ Briefly, *Agrobacterium tumefaciens* GV3101 harboring the recombinant pTRV2 plasmid and GV3101 was mixed with the pTRV2 plasmid in a ratio of 1:1, and then infiltrated into the cotton cotyledons of resistant cultivar ND601, the empty TRV vector (TRV:*00*) was used as the negative control. The experiments were repeated three times independently with at least 35 plants for each treatment. At 25 dpi, the VW symptoms of plants were classified into five grades, the DI was calculated as previously described.^[^ ^57^ ^]^

### Generation and Disease Resistance Analysis of Transgenic Arabidopsis

Full‐length coding sequence of *GhPSB27*, *GhCDSP32*, *GhHDA15*, *GhSRT1*, and *GhHAC2* were cloned from resistant cotton cultivar NDM8 and inserted into the pGreen vector. The site‐directed mutagenesis of *GhPSB27* and *GhCDSP32* were conducted using the method of whole gene synthesis. Mutants were generated by changing the AAA/G (amino acid: K) into CGC/G (amino acid: R), ACC/G (amino acid: T), and GAA/G (amino acid: E) to mimic unmodified, Khib, and Ksuc state, respectively. The recombinant plasmid was transformed into WT Arabidopsis via floral‐dip method. The transformants were screened by BASTA, and the homozygous transgenic lines were identified by PCR and Western blotting (using anti‐HA tag antibody). For each gene, three homozygous lines with high transcription levels were used for the following VW resistance tests. Three‐week‐old Arabidopsis plants were inoculated with *V. dahliae*, and the degree of VW resistance was calculated at 20 dpi as previously described.^[^ ^60^ ^]^

### Immunoblotting

The protein was extracted as described above. About 30 µg total proteins for each sample was separated by 12% SDS–PAGE and transferred into the PVDF membrane for Khib or Ksuc detected, followed by immunoblotting with a rabbit pan anti‐Khib/Ksuc/Kac/Kcr primary antibody (1:1000 dilution, PTM‐801/PTM‐401/PTM101/PTM502, PTM Bio) which are highly specific for the detection of Khib/Ksuc/Kac/Kcr proteins at lysine sites, and neither cross‐reacts with acetylated proteins nor binds nonspecifically with other proteins.^[^ ^61^ ^]^ For the PsbA (D1 protein), the C‐terminal rabbit antibody (AS05084, Agrisera) was used for immunoblotting. The specific anti‐GhPSB27K72suc antibody was customized by PTM Bio. The peroxidase conjugated goat anti‐mouse secondary antibody (1:3000, CW0103S, CW Bio) was directed against rabbit antibodies. Anti‐Actin/Histone H3 antibody serves as loading control. The SuperSignal West Femto chemiluminescence kit (NCI4106, Pierce) was used to detect the signal. The relative quantified signals were calculated using ImageJ.

### Trichostatin A and Nicotinamide Treatment

Cotton ND601 seedlings at two‐leaf period were sprayed with 0.5 × 10^−6^ m trichostatin A (TSA) or 10 × 10^−3^ m NAM and covered with plastic wrap to maintain high humidity. Leaf tissues were collected from the control, TSA‐, and NAM‐treated plants at 24 and 48 h postsprayed. The samples were frozen in liquid nitrogen for subsequent protein extraction.

### In Vitro de‐Khib/Ksuc Assay

The *GhHDA15* and *GhSRT1* coding sequence (without the N‐terminal SP) was cloned into the pET‐28a(+) vector, subsequent expression and purification of recombinant proteins was performed as previously described.^[^ ^52^ ^]^ The cotton histones were extracted using Plant Histone extraction Kit (Solarbio, Beijing, China) according to the manufacturers’ instructions. Purified recombinant GhHDA15 and GhSRT1 were combined with the extracted total cotton histones and inoculated in the HAT buffer (50 mmol L^−1^ Tris‐HCl, pH 8.5, 137 mmol L^−1^ NaCl, 2.7 mmol L^−1^ KCl, 1 mmol L^−1^ MgCl_2_, and 1 mmol L^−1^ dithiothreitol), at 30 °C for 3 or 6 h. Subsequently, the Khib or Ksuc levels of the mixture were determined by immunoblotting using the pan anti‐Khib/Ksuc antibody. Anti‐Histone H3 antibody serves as loading control.

### Histochemical Staining and Determination of ROS Content

The leaves of Arabidopsis and cotton were collected from the same position of different plants. DAB and NBT were used to detect the O_2_^−^ and H_2_O_2_ deposition, respectively. After decolorization in ethanol, the images were captured under a scanner. About 100 mg of fresh leaves were harvested, and ground into power in liquid nitrogen which were used to detect the contents of O_2_^−^ and H_2_O_2_ using the commercial kit (Beijing Boxbio Science & Technology Co., Ltd.).

### Determination of Photosynthetic Characteristics

The fully expanded sixth rosette leaves of 5‐week‐old Arabidopsis and the second true leaves of 30‐day‐old cotton were used to determine net rate of photosynthesis, intercellular CO_2_ concentration, transpiration rate, and stomatal conductance by using LI‐6400/XT portable photosynthetic measurement system (Lincoln, NE, USA). Leaf chlorophyll index of the same Arabidopsis and cotton leaves were measured by using SPAD meter (SPAD 502 plus, Minolta, Japan).

### Chromatin Immunoprecipitation

Three grams of cotton seedling roots was cross‐linked by 1% formaldehyde in a vacuum and used for chromatin extraction. The chromatin was fragmented to 300–500 bp by ultrasonic DNA Interrupter on an ice bath, then chromatin fragments were incubated with 10 µg pan anti‐Khib (PTM‐801, PTM Bio) coated beads (10001D; Invitrogen/Life Technologies) overnight at 4 °C. Rabbit lgG was used as a negative control. After extensively washing, immunoprecipitated chromatin was de‐cross‐linked and retrieved. The precipitated DNA samples were used for qRT‐PCR analysis.

### qRT‐PCR and ChIP‐qPCR Analysis

Total RNA extracted and the first‐strand cDNA synthesized were performed as previously described.^[^ ^56^ ^]^ qRT‐PCR analysis was performed in 10 µL reactions using the Fast Super EvaGreen qPCR Master Mix (US Everbright Inc., Suzhou, China) with three technical replicates and three biological replicates. The gene‐specific primers were used to analyze the expression. The cotton *GhUBQ14* and Arabidopsis *AtTUB2* were used for endogenous control in qRT‐PCR.

## Author Contributions

Z.M., Y.Z., and X.W. conceived and designed the experiments. Y.Z. performed Khib and Ksuc experiments, and B.C. performed gene function validation. B.C., Z.W., M.J., J.Z., J.L., Y.L., G.W., H.K., Q.C., J.Y., Z.S., Q.G., X.W., J.W., L.W., and G.Z. conducted part of the experiments. B.C. and D.Z. completed the ChIP‐qPCR experiment. B.C. and Y.Z. wrote the manuscript. Z.M. and X.W. revised the manuscript. All authors read and approved the contents of this manuscript.

## Conflict of Interest

The authors declare no conflict of interest.

## Supporting information

Supporting Information

Supplemental Table 1

Supplemental Table 2

Supplemental Table 3

Supplemental Table 4

Supplemental Table 5

Supplemental Table 6

Supplemental Table 7

## Data Availability

The data that support the findings of this study are available in the supplementary material of this article.

## References

[1] J. D. G. Jones , J. L. Dangl , Nature 2006, 444, 323. 17108957 10.1038/nature05286

[2] B. Huot , J. Yao , B. L. Montgomery , S. Y. He , Mol. Plant 2014, 7, 1267. 24777989 10.1093/mp/ssu049 PMC4168297

[3] T. L. Karasov , E. Chae , J. J. Herman , J. Bergelson , Plant Cell 2017, 29, 666. 28320784 10.1105/tpc.16.00931 PMC5435432

[4] C. M. Pieterse , D. Van der Does , C. Zamioudis , A. Leon‐Reyes , S. C. Van Wees , Annu. Rev. Cell Dev. Biol. 2012, 28, 489. 22559264 10.1146/annurev-cellbio-092910-154055

[5] T. M. Nolan , N. Vukasinovic , D. Liu , E. Russinova , Y. Yin , Plant Cell 2020, 32, 295. 31776234 10.1105/tpc.19.00335 PMC7008487

[6] D. Wang , K. Pajerowska‐Mukhtar , A. H Culler , X. Dong , Curr. Biol. 2007, 17, 1784. 17919906 10.1016/j.cub.2007.09.025

[7] J. Ye , T. Zhong , D. Zhang , C. Ma , L. Wang , L. Yao , Q. Zhang , M. Zhu , M. Xu , Mol. Plant 2019, 12, 360. 30853061 10.1016/j.molp.2018.10.005

[8] X. Li , D. L. Yang , L. Sun , Q. Li , B. Mao , Z. H. He , Plant Physiol. 2016, 172, 546. 27378815 10.1104/pp.16.00129 PMC5074604

[9] D. L. Yang , J. Yao , C. S. Mei , X. H. Tong , L. J. Zeng , Q. Li , L. T. Xiao , T. Sun , J. Li , X. Deng , C. Lee , M. Thomashow , Y. Yang , Z. He , S. Y. He , Proc. Natl. Acad. Sci. U. S. A. 2012, 109, E1192. 22529386 10.1073/pnas.1201616109 PMC3358897

[10] W. Li , Z. Zhu , M. Chern , J. Yin , C. Yang , L. Ran , M. Cheng , M. Cheng , M. He , K. Wang , J. Wang , X. Zhou , X. Zhu , Z. Chen , J. Wang , W. Zhao , B. Ma , P. Qin , W. Chen , Y. Wang , J. Liu , W. Wang , X. Wu , P. Li , J. Wang , L. Zhu , S. Li , X. Chen , Cell 2017, 170, 114. 28666113 10.1016/j.cell.2017.06.008

[11] M. Liu , Z. Shi , X. Zhang , M. Wang , L. Zhang , K. Zheng , J. Liu , X. Hu , C. Di , Q. Qian , Z. He , D. Yang , Nat. Plants 2019, 5, 389. 30886331 10.1038/s41477-019-0383-2

[12] X. Sun , Y. Xiang , N. Dou , H. Zhang , S. Pei , A. V. Franco , M. Menon , B. Monier , T. Ferebee , T. Liu , S. Y. Liu , Y. C. Gao , J. Wang , W. Terzaghi , J. B. Yan , S. Hearne , L. Li , F. Li , M. Q. Dai , Nat. Biotechnol. 2023, 41, 120. 36229611 10.1038/s41587-022-01470-4

[13] B. Zhang , X. Liu , Y. Sun , L. Xu , Z. Ren , Y. Zhao , Y. Han , Front. Plant Sci. 2022, 13, 928040. 35903230 10.3389/fpls.2022.928040 PMC9317951

[14] Y. Hong , Z. Wang , X. Liu , J. Yao , X. Kong , H. Shi , J. K. Zhu , Plant Physiol. 2020, 182, 1007. 31776182 10.1104/pp.19.01106 PMC6997674

[15] J. R. Yuan , T. T. Ma , S. L. Ji , B. Hedtke , B. Grimm , R. C. Lin , New Phytol 2022, 235, 1868. 35615903 10.1111/nph.18273

[16] F. Yang , K. Xiao , H. Pan , J. Liu , Front. Plant Sci. 2021, 12, 637853. 33747017 10.3389/fpls.2021.637853 PMC7966814

[17] G. R. Littlejohn , S. Breen , N. Smirnoff , M. Grant , New Phytol. 2021, 229, 3088. 33206379 10.1111/nph.17076

[18] Y. Deng , K. Zhai , Z. Xie , D. Yang , X. Zhu , J. Liu , X. Wang , P. Qin , Y. Yang , G. Zhang , Q. Li , J. Zhang , S. Wu , J. Milazzo , B. Mao , E. Wang , H. Xie , D. Tharreau , Z. He , Science 2017, 355, 962. 28154240 10.1126/science.aai8898

[19] Z. Y. Ma , S. P. He , X. F. Wang , J. L. Sun , Y. Zhang , G. Y. Zhang , L. Q. Wu , Z. K. Li , Z. H. Liu , G. F. Sun , Y. Y. Yan , Y. H. Jia , J. Yang , Z. E. Pan , Q. S. Gu , X. Y. Li , Z. W. Sun , P. H. Dai , Z. W. Liu , W. F. Gong , J. H. Wu , M. Wang , H. W. Liu , K. Y. Feng , H. F. Ke , J. D. Wang , H. Y. Lan , G. N. Wang , J. Peng , N. Wang , et al., Nat. Genet. 2018, 50, 803.29736016

[20] Z. Ma , Y. Zhang , L. Wu , G. Zhang , Z. Sun , Z. Li , Y. Jiang , H. Ke , B. Chen , Z. Liu , Q. Gu , Z. Wang , G. Wang , J. Yang , J. Wu , Y. Yan , C. Meng , L. Li , X. Li , S. Mo , N. Wu , L. Ma , L. Chen , M. Zhang , A. Si , Z. Yang , N. Wang , L. Wu , D. Zhang , Y. Cui , et al., Nat. Genet. 2021, 53, 1385. 34373642 10.1038/s41588-021-00910-2 PMC8423627

[21] Y. Zhang , G. Wang , L. Song , P. Mu , S. Wang , W. Liang , Q. Lin , BMC Genomics 2017, 18, 309. 28427325 10.1186/s12864-017-3698-2 PMC5397794

[22] M. Zhang , F. Q. Tan , Y. J. Fan , T. T. Wang , X. Song , K. D. Xie , X. M. Wu , F. Zhang , X. X. Deng , J. W. Grosser , W. W. Guo , Plant Physiol. 2022, 190, 2519. 36135821 10.1093/plphys/kiac442 PMC9706433

[23] M. Hartl , M. Füßl , P. Boersema , J. Jost , K. Kramer , A. Bakirbas , J. Sindlinger , M. Plöchinger , D. Leister , G. Uhrig , G. Moorhead , J. Cox , M. Salvucci , D. Schwarzer , M. Mann , L. Finkemeier , Mol. Syst. Biol. 2017, 13, 949. 29061669 10.15252/msb.20177819 PMC5658702

[24] K. Zhang , H. Z. Cao , Y. X. Ma , H. L. Si , J. P. Zang , H. Bai , L. Yu , X. Pang , F. Zhou , J. Xing , J. Dong , Front. Plant Sci. 2022, 13, 1000039. 36186065 10.3389/fpls.2022.1000039 PMC9521605

[25] H. Zhou , I. Finkemeier , W. Guan , M. A. Tossounian , B. Wei , D. Young , J. Huang , J. Messens , X. Yang , J. Zhu , M. H. Wilson , W. Shen , Y. Xie , C. H. Foyer , Plant, Cell Environ. 2018, 41, 1139. 29126343 10.1111/pce.13100

[26] X. Chen , Q. Xu , Y. Duan , H. Liu , X. Chen , J. Huang , C. Luo , D. X. Zhou , L. Zheng , J Integr Plant Biol 2021, 63, 1801. 34245484 10.1111/jipb.13149

[27] W. Liu , L. Triplett , X. L. Chen , Annu. Rev. Phytopathol. 2021, 59, 99. 33909479 10.1146/annurev-phyto-021320-010948

[28] L. Dai , C. Peng , E. Montellier , Z. Lu , Y. Chen , H. Ishii , A. Debernardi , T. Buchou , S. Rousseaux , F. Jin , B. R. Sabari , Z. Deng , C. D. Allis , B. Ren , S. Khochbin , Y. Zhao , Nat. Chem. Biol. 2014, 10, 365. 24681537 10.1038/nchembio.1497

[29] S. Zhao , X. Zhang , H. Li , Curr. Opin. Struct. Biol. 2018, 53, 169. 30391813 10.1016/j.sbi.2018.10.001

[30] J. L. Avalos , K. M. Bever , C. Wolberger , Mol. Cell 2005, 17, 855. 15780941 10.1016/j.molcel.2005.02.022

[31] K. Ekwall , T. Olsson , B. M. Turner , G. Cranston , R. C. Allshire , Cell 1997, 91, 1021. 9428524 10.1016/s0092-8674(00)80492-4

[32] L. Song , G. Wang , A. Malhotra , M. P. Deutscher , W. Liang , Nucleic Acids Res. 2016, 44, 1979. 26847092 10.1093/nar/gkw053 PMC4797298

[33] D. Zhao , S. W. Zou , Y. Liu , X. Zhou , Y. Mo , P. Wang , Y. H. Xu , B. Dong , Y. Xiong , Q. Y. Lei , K. L. Guan , Cancer Cell 2013, 23, 464. 23523103 10.1016/j.ccr.2013.02.005 PMC3885615

[34] X. Y. Ye , X. M. Niu , L. P. Gu , Y. H. Xu , Z. M. Li , Y. F. Yu , Z. W. Chen , S. Lu , OncoTargets Ther. 2017, 8, 6984.

[35] J. Theis , M. Schroda , Plant Signaling Behav. 2016, 11, e1218587. 10.1080/15592324.2016.1218587 PMC5058467 27494214

[36] A. Abdelraheem , H. Alassbli , Y. Zhu , V. Kuraparthy , L. Hinze , D. Stelly , T. Wedegaertner , J. Zhang , Theor. Appl. Genet. 2020, 133, 563. 31768602 10.1007/s00122-019-03487-x

[37] J. Wang , L. Zhou , H. Shi , M. Chern , H. Yu , H. Yi , M. He , J. Yin , X. Zhu , Y. Li , W. Li , J. Liu , Science 2018, 361, 1026. 30190406 10.1126/science.aat7675

[38] N. Li , B. Lin , H. Wang , X. M. Li , F. F. Yang , X. H. Ding , J. B. Yan , Z. H. Chu , Nat. Genet. 2019, 51, 1540. 31570888 10.1038/s41588-019-0503-y

[39] P. Wang , L. Zhou , P. Jamieson , L. Zhang , Z. Zhao , K. Babilonia , W. Shao , L. Wu , R. Mustafa , L. Amin , A. Diomaiuti , D. Pontiggia , S. Ferrari , Y. Hou , P. He , L. Shan , Plant Cell 2020, 32, 3978. 33037150 10.1105/tpc.19.00950 PMC7721343

[40] G. Wang , J. Xu , L. Li , Z. Guo , Q. Si , G. Zhu , X. Wang , W. Guo , Plant Biotechnol. J. 2020, 18, 222. 31207065 10.1111/pbi.13190 PMC6920168

[41] Y. Zhang , X. F. Wang , S. Yang , J. N. Chi , G. Y. Zhang , Z. Y. Ma , Plant Cell Rep. 2011, 30, 2085. 21739145 10.1007/s00299-011-1115-x

[42] L. Wang , D. Guo , G. Zhao , J. Wang , S. Zhang , C. Wang , X. Guo , New Phytol. 2022, 236, 249. 35727190 10.1111/nph.18329

[43] Q. Hu , L. Min , X. Y. Yang , S. X. Jin , L. Zhang , Y. Y. Li , Y. Z. Ma , X. W. Qi , D. Li , H. Liu , K. Lindsey , L. F. Zhu , X. L. Zhang , Plant Physiol. 2018, 176, 1808. 29229698 10.1104/pp.17.01628 PMC5813555

[44] Y. Miao , L. Xu , X. He , L. Zhang , M. Shaban , X. Zhang , L. Zhu , Plant J. 2019, 98, 329. 30604574 10.1111/tpj.14222

[45] Y. Zhang , B. Chen , Z. Sun , Z. Liu , Y. Cui , H. Ke , Z. Wang , L. Wu , G. Zhang , G. Wang , Z. Li , J. Yang , J. Wu , R. Shi , S. Liu , X. Wang , Z. Ma , Plant Biotechnol. J. 2021, 19, 2126. 34160879 10.1111/pbi.13650 PMC8486238

[46] A. Abdelraheem , H. Elassbli , Y. Zhu , V. Kuraparthy , L. Hinze , D. Stelly , T. Wedegaertner , J. Zhang , Theor. Appl. Genet. 2020, 133, 563. 31768602 10.1007/s00122-019-03487-x

[47] M. E. Dusenge , A. Duarte , D. A. Way , New Phytol. 2018, 221, 32. 29983005 10.1111/nph.15283

[48] D. N. Wilson , J. H. Doudna Cate , Perspect. Biol. 2012, 4, a011536. 10.1101/cshperspect.a011536 PMC3331703 22550233

[49] G. Huang , Y. Xiao , X. Pi , L. Zhao , Q. Zhu , W. Wang , T. Kuang , G. Han , S. F. Sui , J. R. Shen , Proc. Natl. Acad. Sci. U. S. A 2021, 118, e2018053118. 33495333 10.1073/pnas.2018053118 PMC7865125

[50] D. M. Ford , R. Shibles , D. E. Green , Crop Sci. 1983, 23, 517.

[51] M. Gutierrez‐Rodriquez , M. P. Reynolds , A. Larqué‐Saavedra , Bull. ‐ Commonw. Bur. Pastures Field Crops 2000, 66, 51.

[52] T. R. Sinclair , T. W. Rufty , R. S. Lewis , Trends Plant Sci. 2019, 24, 1032. 31488354 10.1016/j.tplants.2019.07.008

[53] Z. H. He , S. Webster , S. Y. He , Curr. Biol. 2021, 32, R634. 10.1016/j.cub.2022.04.070 35728544

[54] J. A. Foley , N. Ramankutty , K. A. Brauman , E. S. Cassidy , J. S. Gerber , M. Johnston , N. D. Mueller , C. O'Connell , D. K. Ray , P. C. West , C. Balzer , E. M. Bennett , S. R. Carpenter , J. Hill , C. Monfreda , S. Polasky , J. Rockström , J. Sheehan , S. Siebert , D. Tilman , D. P. M. Zaks , Nature 2011, 478, 337. 21993620 10.1038/nature10452

[55] S. Kumar , A. Jain , A. P. Shukla , S. Singh , M. Masud , Math. Probl. Eng. 2021, 2021, 1790171.

[56] B. Chen , Y. Zhang , Z. Sun , Z. Liu , D. Zhang , J. Yang , G. Wang , J. Wu , H. Ke , C. Meng , L. Wu , Y. Yan , Y. Cui , Z. Li , L. Wu , G. Zhang , X. Wang , Z. Ying , Plant J. 2021, 107, 831. 34008265 10.1111/tpj.15349

[57] B. Chen , Y. Zhang , J. Yang , M. Zhang , Q. Ma , X. Wang , Z. Ma , Crop J. 2021, 9, 823.

[58] M. Wang , L. Tu , D. Yuan , D. Zhu , C. Shen , J. Li , F. Liu , L. Pei , P. Wang , G. Zhao , Z. Ye , H. Huang , F. Yan , Y. Ma , L. Zhang , M. Liu , J. You , Y. Yang , Z. Liu , F. Huang , B. Li , P. Qiu , Q. Zhang , L. Zhu , S. Jin , X. Yang , L. Min , G. Li , L. L. Chen , H. Zheng , et al., Nat. Genet. 2019, 51, 224. 30510239 10.1038/s41588-018-0282-x

[59] X. Q. Gao , T. Wheeler , Z. H. Li , C. M. Kenerley , P. He , L. B. Shan , Plant J. 2011, 66, 293. 21219508 10.1111/j.1365-313X.2011.04491.x PMC3078967

[60] B. L. Zhang , Y. W. Yang , T. Z. Chen , W. G. Yu , T. L. Liu , H. J. Li , X. H. Fan , Y. Z. Ren , D. Y. Shen , L. Liu , D. L. Dou , Y. H. Chang , PLoS One 2012, 7, e51091. 23251427 10.1371/journal.pone.0051091 PMC3519487

[61] L. Zheng , C. Li , X. Ma , H. Zhou , Y. Liu , P. Wang , H. Yang , Y. Tamada , J. Huang , C. Wang , Z. Hu , X. Wang , G. Wang , H. Li , J. Hu , X. Liu , C. Zhou , Y. Zhang , Nucleic Acids Res. 2021, 49, 7347. 34165567 10.1093/nar/gkab536 PMC8287917

