# Supplementary material for: Lysine 2‐Hydroxyisobutyrylation‐ and Succinylation‐Based Pathways Act Inside Chloroplasts to Modulate Plant Photosynthesis and Immunity
Source: Adv Sci (Weinh). 2023 Jul 26;10(27):2301803. doi: 10.1002/advs.202301803 (PMC10520639; doi:10.1002/advs.202301803)
Supplement: Supplementary file 1 — Supporting Information [file ADVS-10-2301803-s005.pdf]

ADVANCED SCIENCE
Open Access

Supporting Information

for *Adv. Sci.*, DOI 10.1002/advs.202301803

Lysine 2-Hydroxyisobutyrylation- and Succinylation-Based Pathways Act Inside Chloroplasts to Modulate Plant Photosynthesis and Immunity

Bin Chen, Zhicheng Wang, Mengjia Jiao, Jin Zhang, Jie Liu, Dongmei Zhang, Yanbin Li, Guoning Wang, Huifeng Ke, Qiuxia Cui, Jun Yang, Zhengwen Sun, Qishen Gu, Xingyi Wang, Jinhua Wu, Liqiang Wu, Guiyin Zhang, Xingfen Wang, Zhiying Ma* and Yan Zhang**

Supporting Information

Figure S1 Sample repeatability test and KEGG analysis of Khib protein. (a) Box plot of Relative Standard Deviation (RSD) distribution of three repeated samples using quantified Khib proteins. CK, water treatment; Vd, *V. dahliae* infection. (b) KEGG pathway-based enrichment analysis of the Khib proteins in cotton.

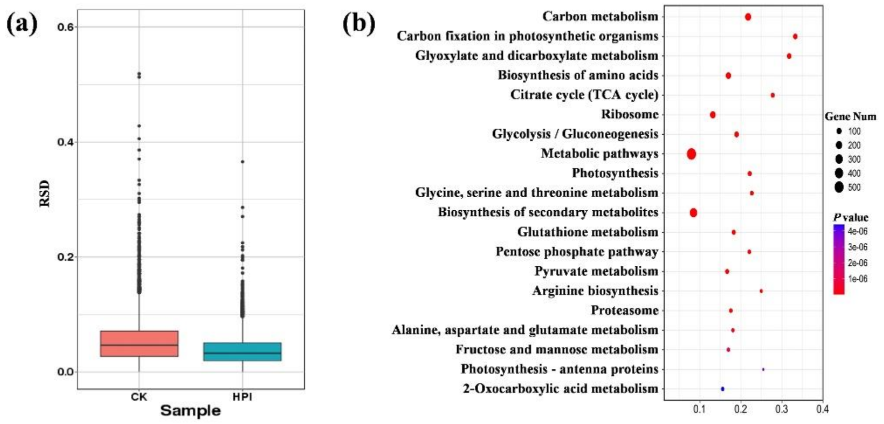

Figure S2 Sample repeatability test and KEGG analysis of Ksuc protein. (a) Box plot of Relative Standard Deviation (RSD) distribution of three repeated samples using quantified Khib proteins. CK, water treatment; Vd, *V. dahliae* infection. (b) KEGG pathway-based enrichment analysis of the Ksuc proteins in cotton.

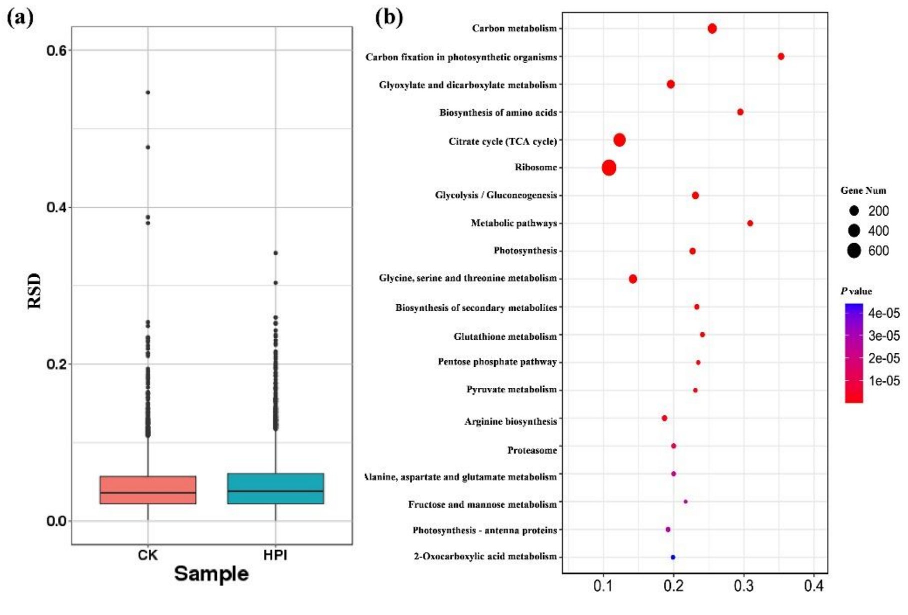

Figure S3 The expression levels of PR, SA and JA genes at 12 hours post-inoculation (hpi) based on transcriptomic data. The relative transcription levels of seven PR-related (a), six SA-related (b)

and six JA-related (c) genes in the infected cotton roots and control. PR, pathogenesis-related gene; ICS1, isochorismate synthase 1; PAD4, phytoalexin deficient 4; PAL, phenylalanine ammonia-lyase; NPR1, nonexpressor of pathogenesis-related genes 1; LOX, lipoxygenase; AOS, allene oxide synthase; MYC2, transcription factor MYC2; AOC, allene oxide cyclase; COI1, coronatine-insensitive protein 1.

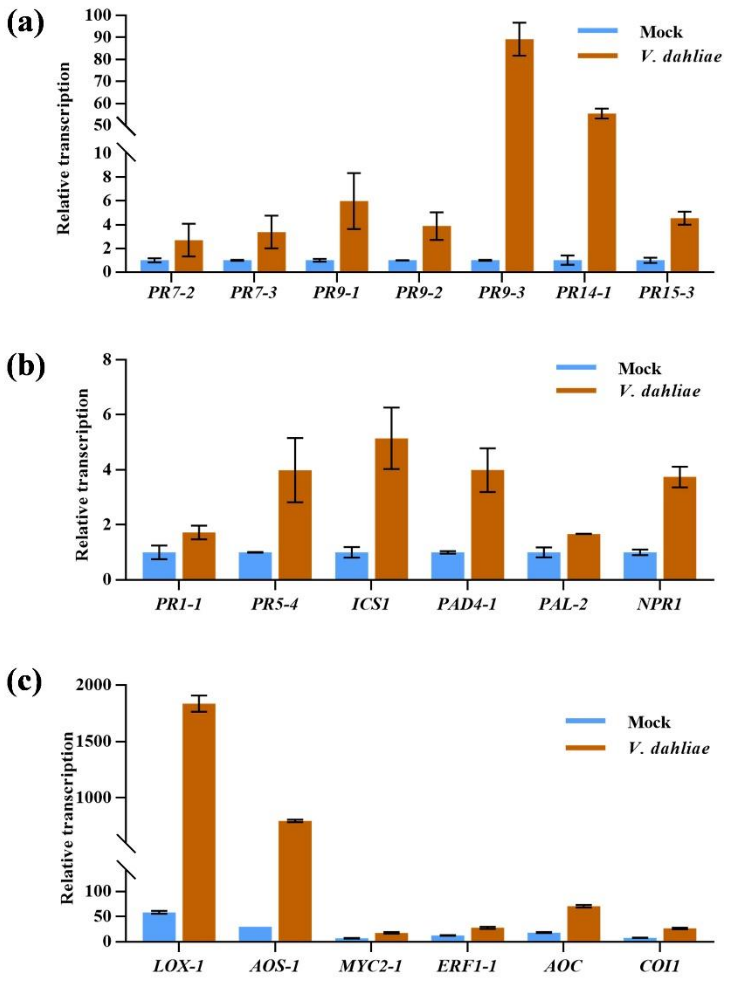

Figure S4 Silencing efficiency of putative Khib/Ksuc "Writer" and "Eraser" detected by qRT-PCR.

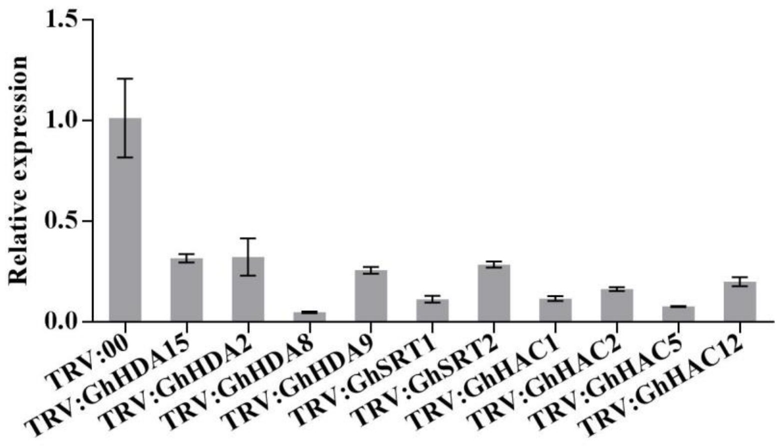

Figure S5 Immunoblotting analysis of total protein acetylation and crotonylation in the TRV:*00* (CK), silenced *GhHDA15* (TRV:*GhHDA15*) and *GhSRT1* (TRV:*GhSRT1*) cottons. (a) Lysine acetylation levels analysis in the silenced and CK cottons by immunoblotting. Anti-Actin antibody serves as loading control. (b) Lysine crotonylation levels analysis in the silenced and CK cottons by immunoblotting. Anti-Actin antibody serves as loading control.

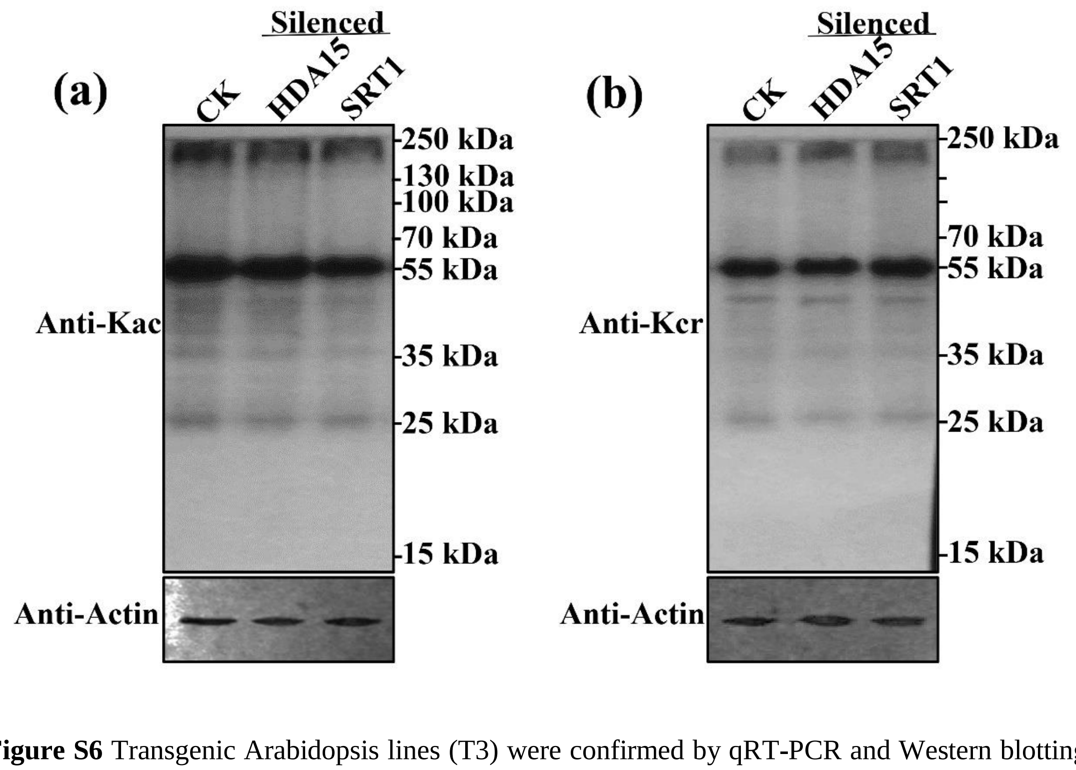

Figure S6 Transgenic Arabidopsis lines (T3) were confirmed by qRT-PCR and Western blotting. (a) Expression of *GhPSB27* or mutant *GhPSB27* in WT and transgenic Arabidopsis by qRT-PCR. Arabidopsis *AtTUB2* was used as an endogenous control. (b) Identification of WT and

homozygous Arabidopsis using anti-HA tag antibody, anti-Actin antibody serves as loading control.

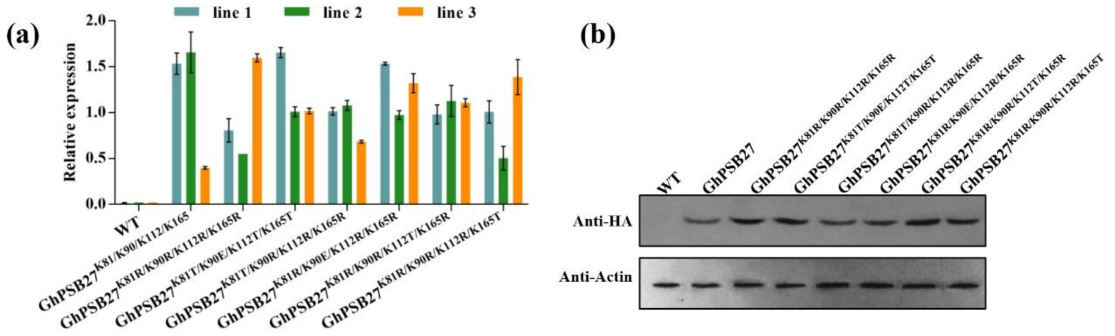

Figure S7 Effects of Khib and Ksuc modified GhPSB27 on Arabidopsis and cotton. (a-d) Intercellular CO_2 concentration, stomatal conductance, transpiration rate, and SPAD value of the WT and transgenic Arabidopsis. (e-h) Intercellular CO_2 concentration, stomatal conductance, transpiration rate, and SPAD value of of three-week-old CK (empty vector, TRV:*00*) and *GhPSB27* silenced (TRV:*GhPSB27*) plants.

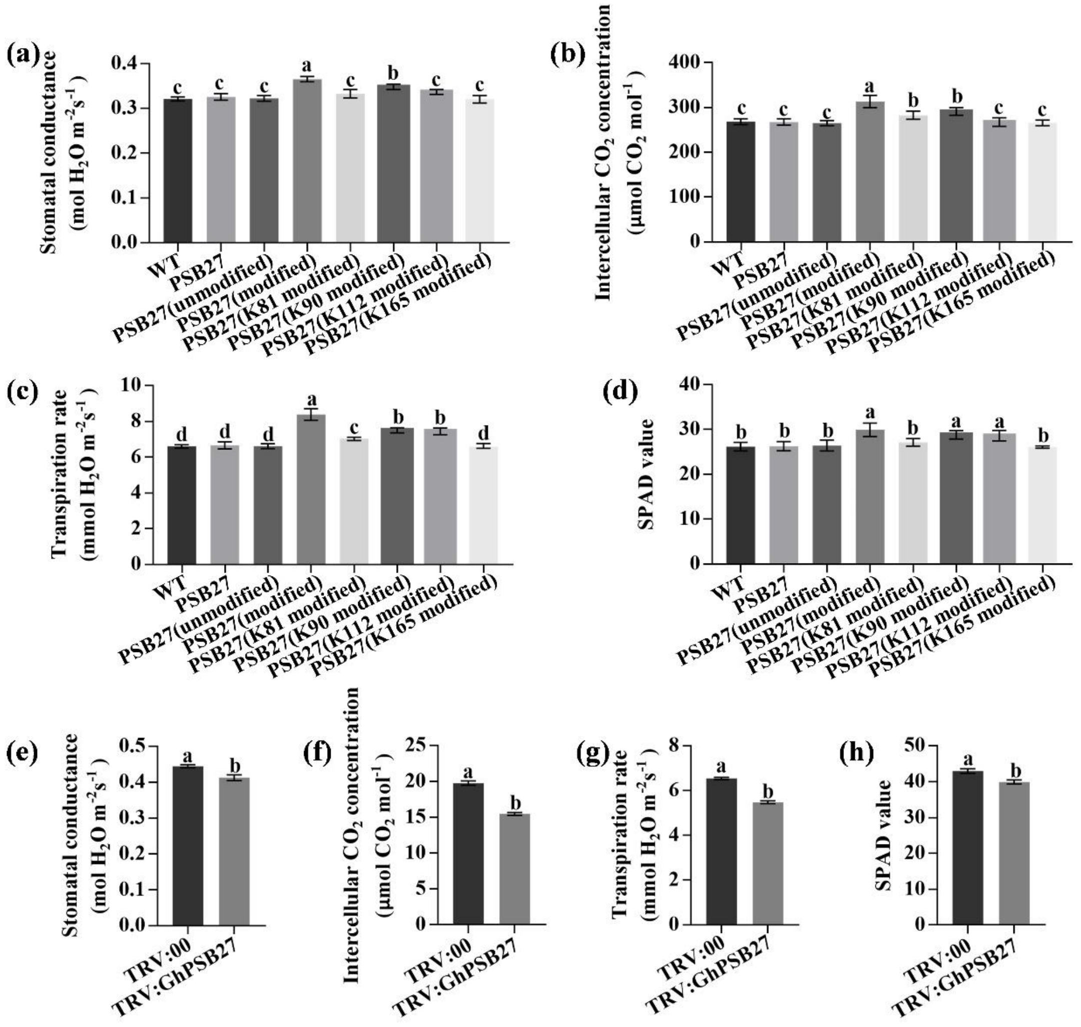

Figure S8 Fragments Per Kilobase of exon model per Million mapped fragments (FPKM) values of *GhHDA15* in four resistant (AustraliaSiV2, Xinmian33B, NDM8 and NDM7) and two susceptible (Handan333 and Xiangmian18) cotton cultivars challenged by *V. dahliae*.

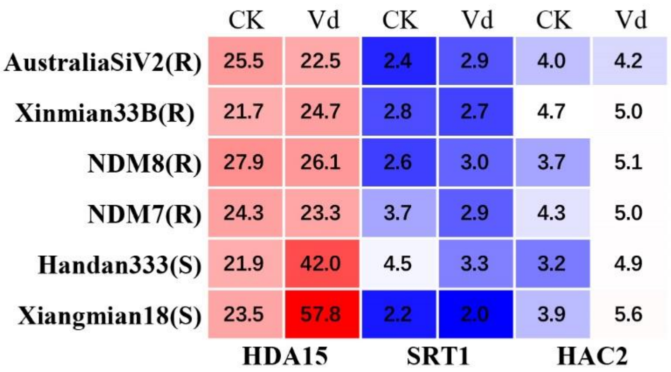

Table S1 Modification site information of Khib proteins in cotton.

Table S2 Gene ontology enrichment of Khib proteins in cotton.

Table S3 Modification site information of cotton Ksuc proteins.

Table S4 The differentially modified Khib proteins in cotton infected with *V. dahliae*.

Table S5 The differentially modified Ksuc proteins in cotton infected with *V. dahliae*.

Table S6 The 21 proteins contain both lysine Khib and Ksuc differently modified sites.

Table S7 Primers used in this study.
